# A New Model to Calibrate a Reference Standard for Bovine Tuberculin Purified Protein Derivative in the Target Species

**DOI:** 10.3389/fvets.2018.00232

**Published:** 2018-10-03

**Authors:** Klaas Frankena, Liesbeth Jacobs, Tonny van Dijk, Margaret Good, Anthony Duignan, Mart C. M. de Jong

**Affiliations:** ^1^Adaptation Physiology Group, Department of Animal Sciences, Wageningen University, Wageningen, Netherlands; ^2^Quantitative Veterinary Epidemiology Group, Department of Animal Sciences, Wageningen University, Wageningen, Netherlands; ^3^Thermofisher Scientific, Prionics Lelystad B.V., Lelystad, Netherlands; ^4^Independent Researcher and Private Consultant, Dun Laoghaire, Ireland; ^5^Department of Agriculture, Food and the Marine, Dublin, Ireland

**Keywords:** *mycobacterium bovis*, tuberculin, bovine international standard, new reference standard, potency estimation, guinea pigs, cattle

## Abstract

Since 1986, use of a Bovine International Standard (BIS) for bovine tuberculin has been required to ensure national and international uniformity regarding the potency designation of bovine tuberculin Purified Protein Derivative (PPDb) preparations produced by multiple manufacturers. The BIS is the unique golden standard in the guinea pig potency assay, representing 100% potency, where potencies of production batches are calculated as relative potencies in comparison with the potency of the BIS which was set at 32,500 international Unit (IU) per mg. The stock supply and lifetime of the BIS is limited.The aim of this study was to develop a model to determine the potency of a newly produced in-house Reference Standard (RS) for PPDb with great accuracy in the target species (cattle) and to prove its precision and accuracy in the guinea pig potency test. First simulations were done to estimate the required number of cattle needed. Then, 30 naturally bTB infected cattle were subjected to a tuberculin skin test using multiple injections of both the RS and the BIS. Both were applied randomly in the same volume and concentration (1 dose). The potency of the RS against the BIS was directly derived from the least square means (LSMEANS) and was estimated as 1.067 (95% CI: 1.025–1.109), equal to a potency of 34,700 ± 1,400 IU/mg. In six guinea pig potency assays the RS was used to assign potencies to production batches of PPDb. Here, precision and accuracy of the RS was determined according to the parallel-line assay. Relative potencies were estimated by exponentiation of the common slope. The corresponding 95% confidence intervals were obtained according to Fieller's theorem. In sensitized guinea pigs, the relative potency of the RS against the BIS was 1.115 (95% CI: 0.871–1.432), corresponding to an absolute potency of 36,238 IU/mg (95% CI: 28,308–46,540).In conclusion: the method used to determine the potency of the RS against the BIS in naturally bTB infected cattle, resulted in a highly accurate potency estimate of the RS. The RS can be used in the guinea pig test to assign potencies to PPDb production batches with high precision and accuracy.

## Introduction

Bovine tuberculosis (bTB) is a zoonotic livestock infection most frequently caused by the bacterium *Mycobacterium bovis* which is often found to be endemic in cattle but which can infect several species of mammals and also marsupials. *Mycobacterium bovis*, belongs to a group of related Mycobacterium species, known as the *Mycobacterium tuberculosis* complex (MTBC), members of which cause tuberculosis in humans and multiple animal species including bovines (1, 2). TB in bovines is a chronic disease characterized by granulomatous lesions in multiple parts of the body and clinical signs such as coughing, reduced milk production etc. Infections can remain subclinical for many years, even if multiple organs are affected (3, 4).

Throughout the last century, extensive control programs resulted in eradication of bTB in many countries, including most EU Member States, Australia, Canada, Switzerland and many states in the USA. Important control measures in cattle include regular and systematic testing of cattle herds, compulsory slaughter of test-positive animals, movement restrictions out of infected herds and post-mortem slaughterhouse surveillance (5–7). However, bTB is still present in various countries, especially in those where a wildlife reservoir of *M. bovis* is present (8). To control and eradicate bTB, multiple tests have been developed to detect infected cattle. The widely used tuberculin skin tests are based on the development of a delayed type hypersensitivity reaction in cattle infected by a MTBC after an intradermal injection with bovine (*M. bovis*) tuberculin Purified Protein Derivative (PPDb) (9, 10). Details about the skin test are in Annex B Directive 64/432/EEC (10) and in OIE guidelines (9).

Since the development of the tuberculin skin tests in cattle, various companies worldwide commenced production of PPDb. To ensure national and international uniformity regarding the potency designation of the PPDb preparations it was essential to define a bovine tuberculin standard. In 1986, after multiple assays in both cattle and guinea pigs, the Bovine International Standard (BIS) was officially established by the World Health Organization (WHO) as the standard for PPDb. The BIS is freeze-dried and stored in glass ampoules, each ampoule containing 1.8 mg of PPDb. Based on the results of an international collaborative study, organized by the WHO, the activity of the contents of each ampoule was defined as 58,500 IU of PPDb (11, page 20), hence the ampoule contained 32,500 IU/mg PPDb Since 1987, the BIS is internationally distributed, on behalf of the WHO, by the International Laboratory for Biological Standards, Hertfordshire, England (11, 12).

Because the stock of the BIS is limited—and reported as seemingly being at the end of its lifetime due to formation of aggregates in some ampoules (13)—, the International Laboratory for Biological Standards encourages manufacturers of PPDb to produce their own Reference Standard (RS) to be used in the guinea pig and cattle potency test. The aim of this study was to determine the potency of a new RS for PPDb with great accuracy in the target species (cattle) and to prove its precision and accuracy in the guinea pig potency test, the prescribed release test for PPD. The new RS was comparable to the BIS in composition and potency in cattle and guinea pigs. A trial in natural bTB infected cattle was designed and performed to determine the potency of the RS in cattle with great precision and accuracy. In the guinea pig test the RS is used to assign potency to individual production batches of PPDb. The accuracy and precision of the RS in the assignment of the potency to production batches of PPDb in the guinea pig potency test was shown in 6 trials. Additionally, the potency of the new RS was compared with the BIS in sensitized guinea pigs.

## Methods

The study consisted of 2 parts: I. Calibration of the RS against the BIS in naturally bTB sensitized cattle. II. Prove of accuracy and precision of the RS in the guinea pig potency test. Here the RS is used to assign potency to individual production batches of PPDb.

### Production of the reference standard

It was decided by Prionics Lelystad B.V. to produce the RS according to the same formula as was used for the BIS with respect to the *M. bovis* strain, volume of the vials, concentration of protein and buffer. Therefore, 15 L of homogenous bulk of PPDb, derived from *M. bovis AN5*, in glucose-phosphate buffer (R31 medium, Hyclone, UK) was formulated with a final concentration of 1 mg/mL (±0.05 mg). Formulation and sterile filtration was performed using Standard Operating Procedures with the exception of adding phenol to the formulated final product. The phenol concentration, due to the phenol present in the starting material of the concentrated tuberculin PPD, is estimated to be 0.03%. No extra phenol was added to the buffer used to formulate the final product. Phenol can evaporate during the freeze-dry process and is hazardous for the environment. The formulated final product was filled into 6 ml vials each vial containing 1.8 mL (±0.02). Freeze drying was done in a Klee Freeze Dryer using program TUB MSL/WSL (freezing of the samples for 3 h at −35°C; drying under vacuum at −33°C at 8.0E-2 mbar for 112 h; drying at 25°C at 1.0 E-2 mbar for 24 h). After freeze drying the vials were closed, capped, labeled and stored in sealed plastic bags at 2–8°C. Before use the content of a vial with the RS is reconstituted in 1.8 ml of Water For Injection (WFI) containing 0.42% phenol to give an end solution in R31 buffer of 0.45% phenol and 1 mg/mL of PPD.

### Calibration of the RS against the BIS in naturally infected cattle

#### Cattle

The trial was carried out on the Longtown Veterinary Research Farm of the Central Veterinary Research Laboratory of the Department of Agriculture, Food and the Marine (DAFM) in Ireland. Thirty naturally infected steers, all between 14 and 24 months old, were selected from herds in which *M. bovis* infection was confirmed (see Supplementary Material for sample size calculation). Cattle which had given a positive skin response, in the single intradermal comparative tuberculin test (SICCT), i.e., showing an increase at the bovine site equal to 4 mm or more than any increase at the avian site, were selected. The time interval between the SICCT on the farms of origin and the study was at least 60 days. All animals also failed (tested positive in) the gamma interferon (y-IFN) Bovigam® test (Thermofisher Scientific, Lelystad) 2 weeks prior to the study, indicating they were responsive to tuberculin.

#### Trial design and testing procedure in cattle

For a RS to be considered as a valid standard, its potency must be estimated with high precision and accuracy. Therefore, the 95% confidence interval was set as the estimated potency ±10% of that potency.

In cattle, four injection sites at each side of the mid-neck are routinely used to perform a potency assay. Therefore, RS and BIS were applied twice on the left side and twice on the right side of the neck. Within the sides of the neck, the injections were randomly allocated to the four injection sites according to one of the 6 unique combinations. For each side, one of the 6 combinations was randomly selected using PROC SURVEYSELECT of SAS version 9.4 (14).

Four injection sites were marked and clipped, using a battery powered hair clippers, on both sides of the mid-neck of the 30 bovines, followed by measurement of the initial skinfold thickness with a caliper before injection. Subsequently cattle were injected (according to the randomization scheme) with a volume of 0.1 mL of BIS or RS at a concentration of 1.0 mg/ml. Skinfold thickness was measured again at 72 h post-injection and the increase in skinfold thickness developed between 0 and 72h was calculated. Injections and measurements were all performed by the same person (AD).

The cattle study was approved by the Health Products Regulatory Authority (HPRA), Dublin, Ireland (project authorization number: AE19113/P008).

#### Statistical analysis

The differences in skinfold thickness were statistically analyzed at a significance level of 5% with a linear mixed model using the increase in diameter of skin at the injection sites (in mm) as outcome variable [PROC MIXED of SAS (14)]. Estimation method used was restricted maximum likelihood (REML) and variance component was specified as covariance structure. The initial model included tuberculin batch (RS, BIS), side (left, right) and site (1–4) of injection and animal was included as random effect. Relative potency can be derived directly from the least square means (LSMEANS) for both batches and its 95% CI from the 95% CI of the difference in LSMEANS. Next, the potency of the RS was calculated by multiplying the relative potency of the RS with the known potency of the BIS i.e. 32,500 IU/mg.

### Proof of accuracy and precision of the RS in the guinea pig potency test

#### Guinea pigs and sensitization

Dunkin Hartley guinea pigs were obtained from ISO 9001 certified breeders of SPF guinea pigs. The guinea pigs were infected with 0.0008 mg of wet mass of living virulent *M. bovis* of strain AN5 by intramuscular injection of 0.5 ml into the left hind leg of each animal. Infection was 5 weeks prior to the skin test. At the moment of infection, the weight of the guinea pig was between 400 and 600 grams.

#### Trial design and assay procedure

Tuberculin skin tests were performed in six trials (labeled T1 to T6) with each 9 guinea pigs. Per guinea pig four injection sites on both sides of the flanks were available. According to the incomplete balanced Latin square design three PPDb batches can be assayed per trial (production batches and/or standards). Batches (production batches and standards) are assayed in three dilutions which were randomly allocated to the injection sites according to the incomplete balanced Latin square design (15). This design can be analyzed as a parallel-line assay (16).

Five weeks after infection, flanks were shaved and treated with depilatory crème leaving enough space for 4 injection sites on each side. Subsequently each guinea pig received eight injections of 0.2 ml of bovine tuberculin PPD. Syringes were coded making persons involved in the GP trials blind for the precise content of any syringe. Diameters of delayed type hypersensitivity reactions, visible as reddish circles around the injection sites, were measured with calipers between 24 and 28 h later. For details of the procedure for skin testing in guinea pigs, see Annex B Directive 64/432/EEC (10), OIE guidelines (9) and the European Pharmacopeia Monograph 0536.

In the six trials, the potency of 4 production batches of PPDb was determined in the guinea pig potency assay using both the RS and the BIS as standard. The four production batches were respectively: A (batch 102402), B (batch 104008), C (batch 110404), and D (batch 112003). In each potency assay, BIS and RS were included and one of the tuberculin batches A–D. Batch A was used in three potency tests, batches B, C, and D each in one potency test. Furthermore, each PPDb was tested in three dilutions 1:200; 1:1,000 and 1:5,000 (concentrations for the RS and the BIS being 0.005, 0.001, and 0.0002 mg/ml. In general, these concentrations result in well measurable skin reactions and acceptable confidence limits (9). Also, these concentrations are 5-fold dilutions, being equidistant at the log scale.

#### Statistical analysis

The goal of the analysis was to determine whether production batches get comparable potency estimates when assayed against either one of both standards (RS or BIS), additionally the relative potency of the RS to the BIS in guinea pigs was calculated. Guinea pig trials were statistically analyzed using generalized linear mixed models [PROC MIXED of SAS (14)] using diameter of skin reaction (in mm) as the outcome variable and guinea pig was included as random effect. Analysis was performed according to a parallel-line assay, as described by Finney (16), with pairs of tuberculins: Batches A, B, C, or D against either RS or BIS and RS against BIS. The independent variables were tuberculin batch (production batch or standard) (Batch), logarithm of the concentration (Logconc), square of Logconc (Logconc^2^) and the interaction between Batch and Logconc (Batch^*^Logconc). Logconc^2^ was included in the model to assess whether or not significant curvature was present. From the interaction Batch^*^Logconc it can be concluded whether or not both batches show parallel lines. Guinea pig (animal) was included as random effect. The assumption was that the diameter outcomes were directly proportional to the logarithm of the tuberculin concentration. For assaying the RS against the BIS a pooled analysis of all six trials was performed. Therefore, the variable Trial was added to the model as fixed effect, as well as the interaction between Batch and Trial. Insignificant variables (*p* > 0.05) were removed from the model using backward model building, except for Batch and Logconc, and Trial in case of the pooled analysis.

Relative potencies were estimated by exponentiation of the common slope. The corresponding 95% confidence intervals were obtained, according to Fieller's theorem (16) which is especially suited for interval estimation of ratios (17) using PROC IML of SAS (14). Finally, the relative potencies of the four additional tuberculin batches against RS were converted into actual potencies by multiplication with the potency estimate of RS from the cattle trial (which appeared to be 34,700 IU/mg). The relative potency of the RS against the BIS was converted to actual potency (expressed in IU) by multiplication with 32,500 IU which is the potency of BIS.

According to the regulations of the European Commission (10), the OIE (9) and the monograph 01/2008 /0536 of the European Pharmacopeia^1^, potency testing of PPDbs in guinea pigs is only valid when the confidence limits are between 50 and 200% of the estimated potency. Furthermore, the estimated potency is not less than 66% and not more than 150% of the stated potency (9).

Guinea pig trials were approved by the Animal Ethic Committee (DEC) of the Animal Science Group of Wageningen University & Research (registration number 1625085300).

## Result

### Cattle trial

#### Descriptive analysis

In total 240 observations were available, half of which were observations on RS injection sites and the other half on BIS injection sites. The average increase in skinfold thickness (between 0 and 72 h) was 6.88 mm (SD 2.81) and 6.48 mm (SD 2.61) for RS and BIS respectively.

#### Potencies

Statistical analysis showed that the variables Batch (*p* = 0.01) and Site (*p* < 0.001) were significantly related to the increase in skinfold thickness. LSMEANS for RS and BIS were 6.90 and 6.46 mm, respectively. The relative potency of RS against BIS (with stated potency of 32,500 IU/mg) was therefore estimated as 6.89540/6.4630 = 1.0669 (95% CI: 1.016–1.118) and the absolute potency of RS is then 1.067^*^32,500 = 34,674 IU/mg (95% CI: 33,020–36,335) or roughly 34,700 ± 1,650. This CI is smaller than the anticipated ±3,000 used in the simulation which is due to smaller variations between bovines (2.5 mm where 3.0 mm assumed) as well as within bovines (1.3 mm where 2.0 mm assumed). In the final model, the intra-class correlation due to the random bovine effect was 0.77.

### Guinea pig trials

#### Descriptive analysis

**Figure 1:**
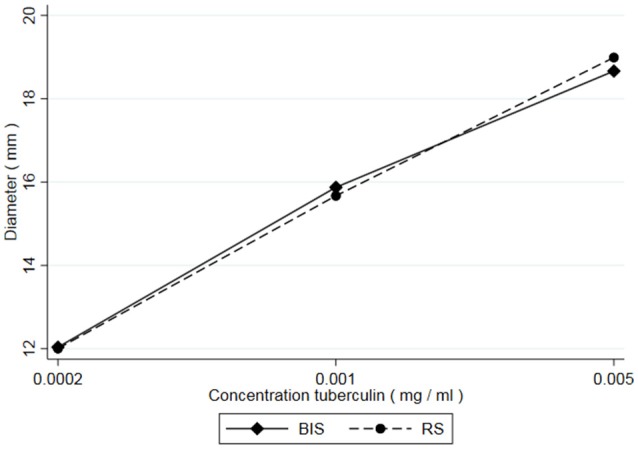
Average skin responses (diameter) of guinea pigs (*n* = 54) after injection with 3 concentrations (equidistant on a log scale) of Bovine International Standard (BIS) and a Reference Standard (RS).

From all injections administered (*n* = 432), 34 resulted in a zero-response (diameter 0.00 mm). All originating from the lowest dose in the potency test (0.0002 mg/ml concentration). From historical data it is known that the lowest dose in the potency test can generate a zero-response. These were treated as missing values and excluded from the analyses because there was no response to the lowest concentration of PPD. Besides that, inclusion of the zero-responses made the distribution of the outcome variable (diameter) non-Gaussian, preventing the valid use of generalized linear mixed models. Additionally, measurements of 36 skin reactions were labeled “weak” meaning they were measured with less accuracy due to an unclear distinction between the reddish hypersensitivity reaction and the normal skin. However, these measurements were not excluded from the analysis, as no clear decision rules exist when to exclude such observations. Table 1 shows the average skin responses of all tuberculins as well as the corresponding minima, maxima and standard deviations. Figure 1 shows the average responses of RS and BIS over the 6 trials.

**Table 1:** Skin response (mm) of guinea pigs after injection with three concentrations of Bovine International Standard (BIS), Reference Standard (RS) and for production batches (A–D) using an incomplete balanced Latin square design; for RS and BIS data of six trials were pooled, for tuberculin A data of three trials were pooled.

**Tuberculin batch**	**Concentration (mg/ml)**	***N***	**Mean**	**Std Dev**	**Minimum**	**Maximum**
RS	0.0002	34	12.00	2.08	8.11	17.84
	0.001	48	15.70	1.92	10.31	18.97
	0.005	48	18.99	1.88	14.83	23.62
BIS	0.0002	36	12.04	1.81	7.25	16.16
	0.001	48	15.88	1.73	11.59	21.49
	0.005	48	18.67	2.00	14.90	24.75
A	0.0002	23	12.40	2.14	6.94	15.36
	0.001	24	16.07	1.40	13.58	18.69
	0.005	24	18.40	1.95	14.46	21.74
B	0.0002	4	12.75	3.68	9.20	17.90
	0.001	8	15.04	2.70	11.77	19.56
	0.005	8	20.23	1.65	17.64	22.47
C	0.0002	7	10.95	1.66	8.83	13.35
	0.001	8	15.78	2.37	12.69	20.05
	0.005	8	19.51	1.99	16.38	21.50
D	0.0002	6	12.02	1.63	9.85	14.12
	0.001	8	15.72	2.60	11.41	19.96
	0.005	8	19.69	0.97	18.10	20.85

#### Parallel line assay

The initial model of the single trial analysis included the terms Batch, Logconc, Logconc^2^, Site, and the interaction between Batch and Logconc. The effect of Site was not significant (*p* > 0.05) for any trial and was eliminated from the model. Logconc^2^ was significant for the batch pair (A, BIS) in trial T2 and the interaction term Batch^*^Logconc was significant for (RS, BIS) and (A, RS) in T2 and for (RS, BIS) in T3. Therefore, these trials were deemed invalid and excluded from further analysis. The final models for T1 and T4-T6 included only Batch and Logconc as independent variables (Table 2).

**Table 2:** Estimated relative and absolute potencies (IU/mg) of pairs of tuberculin batches with 95% CI's in six guinea pig trials.

**Trial**	**Batch pair**	**N**	**Rel. pot. (95% CI)**	**Potency (95% CI)**
T1	RS, BIS	48	1.230 (0.757–2.025)	39,975 (24,603–65,813)^*^
	A, BIS	48	1.233 (0.828–1.853)	40,073 (26,910–62,223)^*^
	A, RS	48	1.095 (0.739–1.627)	37,997 (25,643–56,457)^**^
T2	RS, BIS	48	Non-parallel	–
	A, BIS	48	Significant curvature	–
	A, RS	48	Non-parallel	–
T3	RS, BIS	45	Non-parallel	–
	A, BIS	45	0.782 (0.444–1.338)	25,415 (14,430–43,485)^*^
	A, RS	46	1.081 (0.760–1.542)	35,133 (24,700–50,115)^**^
T4	RS, BIS	41	1.132 (0.678–1.893)	39,280 (23,527–65,687)^*^
	C, BIS	43	1.103 (0.710–1.689)	35,848 (23,075–54,893)^*^
	C, RS	44	0.957 (0.639–1.417)	33,208 (22,173–49,170)^**^
T5	RS, BIS	39	0.954 (0.585–1.565)	31,005 (19,013–50,863)^*^
	D, BIS	42	1.181 (0.794–1.756)	38,383 (25,805–57,070)^*^
	D, RS	41	1.189 (0.766–1.827)	41,258 (26,580–63,397)^**^
T6	RS, BIS	41	1.131 (0.745–1.761)	36,785 (24,213–57,233)^*^
	B, BIS	42	0.975 (0.656–1.465)	31,688 (21,320–47,613)^*^
	B, RS	39	0.852 (0.559–1.285)	29,564 (19,397–44,590)^**^
T1, T4, T5, T6	RS, BIS	169	1.115 (0.871–1.432)	36,238 (28,308–46,540)^*^

* *Relative potencies multiplied by 32,500 (potency assigned to BIS)*.

** *Relative potencies multiplied by 34,700 (potency assigned to RS when calibrated against bis in cattle)*.

#### Potencies

Table 2 displays the relative and absolute potencies and corresponding Fieller's 95% CI of RS compared to BIS, based on data of individual trials and of the four valid trials (T1, T4-T6) pooled. Potencies were not significantly different between RS and BIS because 1.0 is included in all confidence intervals of the relative potency of RS against BIS. The estimated potency of RS based on analysis of the pooled valid trials was 1.115^*^32,500 = 36,238 IU/mg (95% CI: 28,308–46,540).

Table 2 also shows the potency estimates of tuberculin batches A, B, C, and D against RS and BIS. These potency estimations were included to check whether the potency estimations of these additional tuberculin batches against RS were more or less similar to the potency estimations against BIS, which will be the case if the potency estimate of RS as found in cattle is valid. Potencies estimated using RS differ between +2,900 (trial 5, batch B) to −2,700 (trial 4, batch D) compared to BIS. The overall effect of trial was not significant (*p* = 0.16) and also no significant differences were present between individual trials (all *p*-values > 0.16).

## Discussion

Dobbelaer et al. (18) stated that potency estimations in guinea pigs can differ significantly from the potencies in the natural host. Therefore, a cattle trial was designed, performed and analyzed to assign a potency to a reference standard (RS) PPDb. The suitability of the new RS as a *M. bovis* reference standard to assign potency to individual production batches of PPDb was assessed in the guinea pig potency test, the prescribed release test for PPD^1^. Data from tuberculin skin tests in naturally bTB infected cattle and *M. bovis* infected guinea pigs were used to determine the potency of RS compared to the potency of BIS.

To obtain an unbiased potency estimation of the RS, any interference with the potency estimation by the inclusion of other tuberculin batches or tuberculin concentrations, which are not used in practice, should be avoided. Therefore, the cattle trial solely included RS and BIS, in only one dose of 0.1 ml of 1 mg/ml (which is the standard dose of injection in the field) (18).

In the cattle trial, the potency of RS was slightly higher than the potency of BIS. When rounded to the nearest hundred, the potency estimate of RS was 34,700 IU/mg, indicating a difference of 6.8% compared to the potency of BIS.

In guinea pig trials, commonly two test tuberculins are assayed against a standard tuberculin and the common slope of these three tuberculins is then used to estimate the relative potencies of the test tuberculins against the standard tuberculin. However, the most unbiased estimation of the potency of a tuberculin should be solely based on observations of one tuberculin against the standard and by that preventing any influence of the third batch. Therefore, we applied pairwise estimations of potencies, i.e., RS against BIS and of production batches against either RS or BIS. The potency of RS was estimated at 36,238 IU/mg, indicating a difference of 10.3% compared to the potency of BIS. The relative potency estimate of batch A against BIS in trial T3 (TUB 13/009B_Ba) is remarkably lower compared to T1 (0.782 vs. 1.233, *p*-value of trial 0.06) while the relative potency of batch A against RS was very similar (1.081 vs. 1.095) (Table 2). This could be due to an aberrant quality of BIS in the particular ampoule used in trial T3. It is well known that the quality of BIS is decreasing after 30 years of storage. The relative potencies of the four production batches were somewhat lower against RS than against BIS in 3 out of the 4 valid trials.

The cattle model as described in this paper is shown to be an excellent model for precise estimation of the potency of a new RS. Therefore, it is highly recommended to determine the potencies of a new bovine RS in the natural host, i.e., in naturally bTB infected cattle.

However, according to the European Pharmacopeia^1^ the guinea pig potency test is the prescribed release test for production batches of bovine tuberculin PPD. Therefore, it was needed to show the suitability of the new RS needed in the guinea pig model as well. Our results are in accordance with the hypothesis of Dobbelaer et al. (18) that homologous tuberculins result in equal potencies in guinea pigs and in cattle. Indeed, the BIS, the RS and the four production batches used in this study are homologous tuberculins.

## Author contributions

MdJ, KF, LJ, and TvD: conceptualization. KF, LJ, TvD, MG, and AD: data curation. KF and MdJ: formal analysis. MG, LJ, and MdJ: supervision. KF: Writing—original draft preparation.

### Conflict of interest statement

LJ and TvD were employed by company Thermo Fisher, Prionics Lelystad B.V., Lelystad, The Netherlands. The remaining authors declare that the research was conducted in the absence of any commercial or financial relationships that could be construed as a potential conflict of interest. The handling editor declared a past co-authorship with one of the authors KF.

## Footnotes

^1^European Pharmacopeia 9.0. *Monograph 01/2008/0536*.
