# Supplementary material for: A New Model to Calibrate a Reference Standard for Bovine Tuberculin Purified Protein Derivative in the Target Species
Source: Front Vet Sci. 2018 Oct 3;5:232. doi: 10.3389/fvets.2018.00232 (PMC6178890; doi:10.3389/fvets.2018.00232)
Supplement: Supplementary file 1 [file Data_Sheet_1.docx]

Supplementary material

##

## Simulation for Sample Size Determination in Trials for the Development of a Reference Standard in Cattle

Introduction

Potency estimation of production batches of bovine tuberculin PPD is routinely performed in cattle in Ireland. In these trials usually 2 dilutions of 4 tuberculin batches are applied in a Latin square design. Dilutions are either ‘full’ (1/1) or 1/5, where the 1/1 concentration is close to what is used in field testing of cattle. A parallel line assay (PLA) is then used to estimate the potency of a batch of tuberculin relative to the Irish standard. The data of such a trial will allow estimation of (i) the potency of the new batch and (ii) the required dilution needed to achieve a specific potency.

However, for creating a reference standard only the 1/1 concentration of tuberculin will suffice because only in that case an estimate of the potency is required. Using two (or more) concentrations is not desired as that may influence the potency considerably. A retrospective analysis of 18 trials performed in Ireland (including in total 46 different batches of tuberculin applied in 2 concentrations, 1/1 and 1/5 concentration) showed that potency estimates using only the 1/1 concentration was at average 26% (median 31%) lower compared to estimates from the parallel line estimation using both the 1/1 and the 1/5 concentration.

Simulation

As bovines will be injected multiple times with the same solution (BIS or RS), the between- and within-bovine variation in skin responses need to be known to estimate the number of animals to be included in the trial. The between-bovine variation was determined from previous (Irish) trials in which the Irish reference standard had been used and it was estimated as 3.0 mm. For estimation of the within-bovine variation no data were available and simulation was performed. For this, data from an Irish trial in which BIS and Irish standard were evaluated using 24 naturally infected steers were available. For each bovine, one true value of BIS induced skinfold difference was taken from the dataset and three additional measurements were generated using a Gaussian distribution with varying SD’s of 0.5 to 3.0 with increments of 0.5 - the 3.0 being as high as the between bovine variation - at a trial size of 24, 30 and 36 animals. Animals were selected, with replacement, from the existing dataset using PROC SURVEYSELECT. For each combination of SD and sample size, 3000 simulations were performed, and the potency and its corresponding 95% CI were estimated using PROC MIXED; next results were averaged over the 3,000 simulations (Table 1). Key outcome is the error of the estimated potency as this error should be limited in order to estimate the potency of the new standard as precise as possible.

**Table 1**. Potency and Error estimation (average of 3,000 simulations) and 95% confidence intervals (LL CI=lower limit CI and UL CI=upper limit CI) of RS against BIS at varying trial sizes and varying within cow variation (SD) of each tuberculin (SAS, PROC MIXED).

|  |  |  |  |  |  |  | Rounded to nearest 500 | | |
| --- | --- | --- | --- | --- | --- | --- | --- | --- | --- |
| n | SD | Potency | Error | LL CI | UL CI |  | Potency | LL CI | UL CI |
| 24 | 0.5 | 33,279 | 2,495 | 30,784 | 35,774 |  | 33,500 | 31,000 | 36,000 |
| 24 | 1.0 | 33,336 | 2,782 | 30,554 | 36,118 |  | 33,500 | 30,500 | 36,000 |
| 24 | 1.5 | 33,365 | 3,202 | 30,163 | 36,567 |  | 33,500 | 30,000 | 36,500 |
| 24 | 2.0 | 33,387 | 3,711 | 29,676 | 37,098 |  | 33,500 | 29,500 | 37,000 |
| 24 | 2.5 | 33,425 | 4,275 | 29,150 | 37,701 |  | 33,500 | 29,000 | 37,500 |
| 24 | 3.0 | 33,412 | 4,865 | 28,547 | 38,276 |  | 33,500 | 28,500 | 38,500 |
| 30 | 0.5 | 33,291 | 2,301 | 30,990 | 35,592 |  | 33,500 | 31,000 | 35,500 |
| 30 | 1.0 | 33,334 | 2,554 | 30,780 | 35,888 |  | 33,500 | 31,000 | 36,000 |
| 30 | 1.5 | 33,339 | 2,930 | 30,409 | 36,268 |  | 33,500 | 30,500 | 36,500 |
| 30 | 2.0 | 33,270 | 3,374 | 29,896 | 36,644 |  | 33,500 | 30,000 | 36,500 |
| 30 | 2.5 | 33,308 | 3,890 | 29,419 | 37,198 |  | 33,500 | 29,500 | 37,000 |
| 30 | 3.0 | 33,424 | 4,443 | 28,982 | 37,867 |  | 33,500 | 29,000 | 38,000 |
| 36 | 0.5 | 33,244 | 2,146 | 31,098 | 35,398 |  | 33,000 | 31,000 | 35,500 |
| 36 | 1.0 | 33,329 | 2,385 | 30,944 | 35,714 |  | 33,500 | 31,000 | 35,500 |
| 36 | 1.5 | 33,310 | 2,718 | 30,592 | 36,028 |  | 33,500 | 30,500 | 36,000 |
| 36 | 2.0 | 33,302 | 3,136 | 30,166 | 36,438 |  | 33,500 | 30,000 | 36,500 |
| 36 | 2.5 | 33,363 | 3,579 | 29,784 | 36,941 |  | 33,500 | 30,000 | 37,000 |
| 36 | 3.0 | 33,331 | 4,091 | 29,239 | 37,422 |  | 33,500 | 29,000 | 37,500 |

If we assume that the precision of the potency estimate should be not more than ± 10% then this will be achieved at n=24 when SD is between 1.5 and 2.0; for n=30, SD should not exceed 2.0, and for n=36, SD’s can be close to 2.5. Thus, to take either n=24, 30 or 36 depends on the pre-estimate of the within-bovine variation. As the between bovine variation is 3.0 mm, a within- bovine SD of 2.0 mm is a safe choice, so a trial with n=30 should be large enough to achieve an accuracy of ± 3,000.

Table 2. Randomization scheme used to estimate the potency of a reference standard (RS, coded as 2), relative to BIS (coded as 1), in a trial of 30 bovines. L1-L4, R1-R4 are the respective injection sites at the left and right neck side.

| Bovine nr | Random combination  Left | L1 | L2 | L3 | L4 | Random  combination  Right | R1 | R2 | R3 | R4 |
| --- | --- | --- | --- | --- | --- | --- | --- | --- | --- | --- |
| 1 | 1221 | 1 | 2 | 2 | 1 | 2112 | 2 | 1 | 1 | 2 |
| 2 | 1221 | 1 | 2 | 2 | 1 | 2121 | 2 | 1 | 2 | 1 |
| 3 | 2112 | 2 | 1 | 1 | 2 | 2121 | 2 | 1 | 2 | 1 |
| 4 | 2121 | 2 | 1 | 2 | 1 | 1221 | 1 | 2 | 2 | 1 |
| 5 | 1122 | 1 | 1 | 2 | 2 | 2211 | 2 | 2 | 1 | 1 |
| 6 | 1122 | 1 | 1 | 2 | 2 | 2211 | 2 | 2 | 1 | 1 |
| 7 | 2211 | 2 | 2 | 1 | 1 | 2112 | 2 | 1 | 1 | 2 |
| 8 | 1221 | 1 | 2 | 2 | 1 | 1212 | 1 | 2 | 1 | 2 |
| 9 | 1212 | 1 | 2 | 1 | 2 | 1212 | 1 | 2 | 1 | 2 |
| 10 | 2211 | 2 | 2 | 1 | 1 | 2121 | 2 | 1 | 2 | 1 |
| 11 | 2112 | 2 | 1 | 1 | 2 | 2121 | 2 | 1 | 2 | 1 |
| 12 | 1212 | 1 | 2 | 1 | 2 | 1122 | 1 | 1 | 2 | 2 |
| 13 | 1212 | 1 | 2 | 1 | 2 | 2211 | 2 | 2 | 1 | 1 |
| 14 | 2121 | 2 | 1 | 2 | 1 | 1221 | 1 | 2 | 2 | 1 |
| 15 | 2121 | 2 | 1 | 2 | 1 | 1221 | 1 | 2 | 2 | 1 |
| 16 | 1122 | 1 | 1 | 2 | 2 | 2211 | 2 | 2 | 1 | 1 |
| 17 | 2211 | 2 | 2 | 1 | 1 | 2112 | 2 | 1 | 1 | 2 |
| 18 | 1221 | 1 | 2 | 2 | 1 | 1212 | 1 | 2 | 1 | 2 |
| 19 | 1221 | 1 | 2 | 2 | 1 | 1212 | 1 | 2 | 1 | 2 |
| 20 | 2211 | 2 | 2 | 1 | 1 | 1122 | 1 | 1 | 2 | 2 |
| 21 | 2112 | 2 | 1 | 1 | 2 | 2121 | 2 | 1 | 2 | 1 |
| 22 | 2112 | 2 | 1 | 1 | 2 | 2121 | 2 | 1 | 2 | 1 |
| 23 | 1212 | 1 | 2 | 1 | 2 | 1122 | 1 | 1 | 2 | 2 |
| 24 | 2112 | 2 | 1 | 1 | 2 | 2121 | 2 | 1 | 2 | 1 |
| 25 | 2121 | 2 | 1 | 2 | 1 | 1221 | 1 | 2 | 2 | 1 |
| 26 | 1122 | 1 | 1 | 2 | 2 | 1221 | 1 | 2 | 2 | 1 |
| 27 | 1122 | 1 | 1 | 2 | 2 | 2211 | 2 | 2 | 1 | 1 |
| 28 | 2211 | 2 | 2 | 1 | 1 | 2112 | 2 | 1 | 1 | 2 |
| 29 | 1221 | 1 | 2 | 2 | 1 | 1212 | 1 | 2 | 1 | 2 |
| 30 | 1221 | 1 | 2 | 2 | 1 | 1212 | 1 | 2 | 1 | 2 |
